# Supplementary material for: Complexome profiling of the Chlamydomonas psb28 mutant reveals TEF5 as an early PSII assembly factor
Source: Plant Cell. 2025 Mar 17;37(6):koaf055. doi: 10.1093/plcell/koaf055 (PMC12164590; doi:10.1093/plcell/koaf055)
Supplement: koaf055_Supplementary_Data [file koaf055_supplementary_data.zip › psb28_interactive.zip!/plots/intensity/qualityPlots/peptides/FAP354_Cre09.g390763.t1.1.html]

### Cre09.g390763.t1.1

Trivial name: FAP354  
SeqCoverage [median]: 57.70  
Mass: 23.61 kDa  
  
 

| Sequence | MissedCleavage | Mass [Da] | wtIntensity | muIntensity | Score |
| --- | --- | --- | --- | --- | --- |
| AVTTEEGEQFAK | 0 | 1308.62 | 1286.70 | 6745.60 | 157.24 |
| EHGLIFLETSAR | 0 | 1371.71 | 2629.81 | 11964.83 | 97.16 |
| GAAGALLVYDITRR | 1 | 1474.83 | 3685.59 | 0.00 | 89.96 |
| IQDGVFDVSNESYGIK | 0 | 1769.85 | 7525.77 | 12851.41 | 187.45 |
| KIQDGVFDVSNESYGIK | 1 | 1897.94 | 0.00 | 1343.60 | 55.86 |
| LQIWDTAGQESFR | 0 | 1549.75 | 2732.24 | 1831.56 | 124.42 |
| RAVTTEEGEQFAK | 1 | 1464.72 | 317.27 | 0.00 | 49.30 |
| SYAYLFK | 0 | 890.45 | 564.45 | 0.00 | 111.28 |
| TAHNVEEAFINTAK | 0 | 1543.76 | 6397.91 | 12003.63 | 146.58 |
| VGYGAGNAGPQTVKPGEGGAAK | 1 | 1985.00 | 19309.03 | 29608.04 | 163.74 |
| YIIIGDTGVGK | 0 | 1134.63 | 0.00 | 1219.54 | 55.55 |
|  |  |  | 44448.8 | 77568.2 |  |
